# Organizational complexity in big science: strategies and practices

**DOI:** 10.1007/s11229-022-03649-3

**Published:** 2022-05-06

**Authors:** Martina Merz, Helene Sorgner

**Affiliations:** University of Klagenfurt, Sterneckstrasse 15, 9020 Klagenfurt, Austria

**Keywords:** Big Science, Organizational complexity, Bureaucracy, Standardization, Segmentation, Large Hadron Collider

## Abstract

Studies on ‘Big Science’ have shifted our perspective from the complexity of scientific objects and their representations to the complexity of sociotechnical arrangements. However, how scientists in large-scale research attend to this complexity to facilitate and afford knowledge production has rarely been considered to date. In this article, we locate organizational complexity on the level of organizing practices that follow multiple and divergent logics. We identify three strategies of managing organizational complexity, drawing on existing literature on large-scale research as well as own empirical research. The three strategies are: segmenting research infrastructure, introducing elements of bureaucratic governance, and implementing standards and standardization. We illustrate these strategies with examples from our empirical case study on experimental particle physics research at CERN’s Large Hadron Collider. While the strategies we identified help to cope with the complexity of some organizational tasks by dividing, ordering, or mediating between divergent organizational logics, we find that organizational complexity overall is not reduced but rather displaced. We argue that dealing with complexity is a dynamic and ongoing process, which inevitably generates novel organizational complexity.

## Introduction^1^

Philosophers of science have been interested in complexity mainly as a feature of the objects of scientific investigation (e.g., living organisms) or, instead, their representation (e.g., complex systems or models).^2^ In this text, we shift the focus to a perspective that has received little attention by philosophers of science so far: the complexity of the sciences’ sociotechnical arrangements, i.e., their organizational forms and knowledge-making infrastructures. In short, we will label this type of complexity ‘organizational complexity’. This text’s central objective then is to introduce, and raise awareness for, the organizational complexity of science and the social dimensions of knowledge production more generally. We understand organizational complexity to be, at the same time, a feature of the scientists’ sociotechnical environments and an effect of their organizing practices. Thus, we put forward a double argument. On the one hand, we identify three strategies that research collectives employ to attend to organizational complexity. On the other hand, we find that these strategies, in practice, engender further complexity. In other words, complexity is not merely reduced or transformed into a state of simplicity, but reappears in specific ways. To substantiate this double point, we will adopt perspectives and draw on scholarship from the practice approach in Science and Technology Studies (STS) as well as from organizational research, and we will provide examples from our own empirical research.

In particular, our focus will be on the sociotechnical dimensions of *large-scale research*, i.e., research conducted in large teams and typically engaging complicated instrumentation and procedures. In the history and social studies of science, large-scale research of this type has become known as ‘Big Science’ (Galison and Hevly 1992). This notion goes back to the early 1960s where, in the United States and the context of the birth of NASA and the national space program, the term became attached to “projects that required large-scale organization, massive commitments of funds, and complex technological systems” (Capshew and Rader 1992, p. 4). While the notion also refers to the scaling up of science in other respects (e.g., an increase in publications across disciplines, cf. de Solla Price 1963), it will be employed here with reference to the first connotation, which is also in line with how it is used in recent STS scholarship.^3^

In this regard, Big Science refers to scientific endeavors such as collider experiments in particle physics (e.g., at Fermilab or at CERN, the European Laboratory for Particle Physics), telescopes (e.g., the Hubble Space Telescope) or the Human Genome Project. Current collider experiments, such as those at CERN’s Large Hadron Collider (LHC), are typically represented as a story of stacked superlatives concerning the size of machines (accelerators and detectors), the amount of data processed, or the unprecedented number of scientists in a single experiment (more than 3,000 in a collaboration). These striking features are highlighted by physicists, historians, sociologists, and the media alike.

Each of the aforementioned scientific undertakings is ‘big’ in its own way; this diversity would deserve an analysis in its own right. In the present text, we focus on CERN’s LHC experiments that we are studying closely in an ongoing research project^4^: we are interested in the specific socio-material forms of organizing and coordinating that the LHC collaborations and communities devise and adopt to meet the challenges of successfully ‘doing Big Science’. This research focus builds on the core insight of STS research that different forms of knowledge production generate specific organizational forms (Knorr Cetina 1999) which in turn shape the knowledge being produced (e.g., Vertesi 2020). While a certain degree of organizational complexity also pertains to the case of ‘small’ science, distinct organizational challenges arise in contemporary Big Science, as we will show below. The researchers we interviewed devote a significant amount of time to socio-material organizing and coordinating activities concerned with the LHC experiments’ overall complexity. We unpack these activities in detail and demonstrate that even though they may be construed as efforts to reduce the experiment’s complexity, they may introduce other kinds of complexity.

In the remainder of this paper, we first devise a concept of organizational complexity (Sect. 2). Next, we introduce CERN’s LHC experiments, our related case study, and our empirical approach (Sect. 3). We then present three core strategies that researchers, their teams, and communities deploy to deal with Big Science experiments’ organizational complexity (Sect. 4). For this purpose, we combine selected insights from existing STS research into Big Science with our empirical findings. Altogether, we thus put forward a practice-oriented approach to understanding organizational complexity in Big Science.

## Organizational complexity

Our object of study are the practices and social organization associated with scientific knowledge production. What we are studying is thus neither an object of the natural world nor its representation, but the scientists’ generating, interacting with, and ordering their socio-material environments. In other words, we are concerned with the complexity associated with practices of organizing.^5^ Physicists rarely speak of complexity explicitly. ‘Organizational complexity’, as we use it, is thus an analysts’ category. We locate organizational complexity on the level of organizing practices associated with multiple and divergent logics, and argue that the strategies employed to address this pronounced complexity displace and generate, rather than merely reduce, complexity. In the following, we review existing conceptualizations of organizational complexity in organizational research before further specifying our conceptual approach.

In the study of organizations, complexity has been theorized as an effect of both the size and the structure of organizations. Simon (1962) describes complex systems as being “made up of a large number of parts that interact in a nonsimple way” such that “given the properties of the parts and the laws of their interaction, it is not a trivial matter to infer the properties of the whole” (ibid., p. 468). In this sense, all organizations with a large number of members could be considered to be complex. What makes organizations comparatively more or less complex is the degree of differentiation between their constituent elements (Dooley 2002). According to Luhmann (1995), it is not only the increasing number of elements in a system that gives rise to internal complexity but also the selectivity and thus contingency of the relations between individual elements. In other words, a growing number of elements renders it increasingly difficult for the system to realize all the potential relations between its elements, and only select relations are realized. Moreover, the higher the number of elements of a given organization, the more it can get irritated by external influences as the organization has more contact points with its environment (cf. Luhmann 2018, pp. 299–301).

Accordingly, the complexity of Big Science experiments does not only pertain to internal processes but also to the interrelation with their institutional environments, such as political arenas (Hallonsten 2016). In organizational research, the ‘open systems’ view of organizations similarly considers complexity as arising in response to the complexity of an organization’s environment together with the complexity of the organization’s ‘technological core’, the activities to achieve its main goals (Thompson 1967). In his influential analysis, Thompson views organizations as exhibiting structural differentiation to deal with complex environments and tasks. One result of this growing internal complexity is that organizations create units dealing with relatively homogeneous tasks and environments. A related strand of literature studies organizations as ‘complex adaptive systems’. Building on the insights of complexity science, an organization’s complexity is here understood to be an emergent property, an outcome of the actions of interrelated actors in the organization (Anderson 1999).

In the approaches described above, complexity is conceived as a property of organizations and their structure. This family of approaches can be said to defend a notion of ‘ontological complexity’ (Dan-Cohen 2016) applied to organizations.

However, there exists another approach that locates complexity at the representational level, which may broadly be termed the interpretive approach in organization studies (Tsoukas and Hatch 2001). First introduced by Karl Weick (1969), who treats organizations as the outcomes of interactive processes of sense-making, this approach emphasizes that the complexity of an organization depends on how it is understood and represented by its members and observers. Tsoukas and Hatch (2001) go further in their proposal to construe the complexity of any system as an effect of the complexity of the language of its description. The authors argue that a system is the more complex, the larger the number of inequivalent descriptions that can be produced of it; thus, the more complex our vocabulary, the more complex are the objects we describe.

Our own approach to studying organizational complexity builds on this interpretive tradition, yet focuses first and foremost on practices of organizing (which includes sense-making activity). This conception is informed by the practice perspective commonly adopted in STS.^6^ Accordingly, we attend to the actors’ real-life challenges of creating and maintaining organizational order by scrutinizing their everyday efforts and setbacks. The specific notion of organizational complexity we introduce here draws on the work of John Law and Annemarie Mol (Law 1994; Mol and Law 2002).^7^

Law (1994) describes modern organizations to be permeated by different ‘modes of ordering’. A mode of ordering can be identified where actors organize, that is, characterize, sort, evaluate, rank, and represent entities and practices according to some imputable principle or ‘logic’, such as responsible administration, opportunistic enterprise, or the professional norms of the scientific vocation. Importantly, no single order is ever complete or exhaustive; instead, several modes of ordering may be active within the same organization at once, partially overlapping, competing, or depending on one another (ibid.).

Inspired by this account, we understand organizational practices to be multifaceted and capable of enacting different, potentially opposing organizational logics.^8^ In the case of large-scale research, these logics may reflect, for example, different concepts of management and (self-)governance; distinct priorities and expectations of (national) funding agencies, scientists, and relevant publics; historically, culturally or institutionally entrenched relations and norms of interaction; or epistemic norms and conceptions of research quality, which vary between scientific communities. We consider the presence of multiple organizational logics and their associated practices to indicate organizational complexity. While every organization can be said to be complex in this sense, organizations may differ in terms of degree of complexity concerning two features: first, the co-occurrence of multiple organizational logics interacting in non-trivial ways; second, the organization’s recurrent reflexive hum^9^, i.e., the extent to which members reflect upon organizing practices, their principles and effects, and engage more or less intensively in negotiation processes for reorganization.

Although the growth of research facilities is often associated with an increase in (unspecified) organizational complexity (e.g., Perović 2018), we believe that Big Science efforts do not necessarily generate multiple organizational logics and practices, and exhibit self-reflexive ordering to the same degree. As we argue below, contemporary experiments in high-energy physics may, however, be characterized as organizationally complex in the described sense.

We suggest that organizational complexity surfaces in large-scale research where tensions between different organizational logics are implicitly or explicitly addressed, and where organizing itself becomes a focus of scientists’ attention and practices. To identify such moments of tension as well as the strategies scientists develop in response to organizational complexity, we rely on earlier studies on large-scale research as well as insights from our case study on contemporary experimental high-energy physics, the ATLAS collaboration.

## LHC collaborations, case study, and approach

The ATLAS collaboration is the multi-institutional and multi-national team running the eponymous experiment at CERN’s Large Hadron Collider. ATLAS combines several characteristics often associated with complex organizations (see Sect. 2), most notably a numerous, heterogeneous, and distributed constituency of members attending to highly specialized research tasks and cutting-edge technology. On its official website, the collaboration is introduced as “one of the largest collaborative efforts ever attempted in science”.^10^ The page lists various quantitative aspects in terms of membership: 3,000 scientific authors from 183 institutions in 41 countries with 1,200 doctoral students. The collaboration’s governance model is characterized as democratic (“ATLAS elects its leadership”) and participatory (“allows teams to self-manage, and members to be directly involved in decision-making processes”). The organizational structure of ATLAS is said to be reconciling the individual and the collective: on the one hand, work “in small groups”, the free choice of topics, and individual commitment; on the other hand, collective control and ownership (“Any output from the collaboration is shared by all members and is subject to rigorous review and fact-checking processes before results are made public”). The “complete and coherent collaborative effort”, as we read on the webpage, is brought forth by a world-wide constituency of members, which successfully accomplishes a highly sophisticated scientific program relying on very diverse bodies of knowledge and skills as well as technical infrastructure, much of which needed to be developed specifically for the purpose.

The website description of ATLAS suggests that there are various organizational principles and associated practices permeating the collaboration, such as leadership and democratic participation, independent work in small teams and collective control, an ambitious scientific program, and attendance to the technical innovations and infrastructure necessary to support it. We also see a reflexive move in that the website explicitly mentions some organizational challenges (heterogeneity, specialization, and geographical distribution of the constituency), management structures, and organizational values beyond the production of novel scientific results. ATLAS may thus be considered to be a prime example of organizational complexity in Big Science, in the sense we have outlined above.

The ATLAS collaboration is the central case study of our research project on the epistemic and organizational practice of collaborative research at the LHC. Our qualitative research approach is predominantly based on interviews and informal interaction with physicists occupying different positions and functions within the collaboration. Qualitative expert interviews with narrative passages address the specific technical, procedural, and interpretive knowledge that the interviewees have acquired within their individual work contexts (Bogner et al. 2009). Expert interviews are an invaluable resource for reconstructing organizational practices (Merz and Sorgner 2020). Through their continuous engagement in organizing their own research and that of others, members of the ATLAS collaboration have acquired intimate knowledge of organizational practices.

To capture a broad range of these practices, and how they are interpreted by ATLAS members, we carefully selected interview participants representing different perspectives from within the physics community in ATLAS. We have interviewed 30 collaboration members, including a deputy spokesperson, members of various internal committees, and physicists of all career stages (15 senior scientists, 6 post-docs, 9 Ph.D. students). Interviews lasted between 45 min and 3 h. Upon obtaining participants’ written consent, all interviews were recorded and transcribed verbatim. Following a first thematic analysis of the transcripts, we selectively analyzed those instances for close analysis, where organizing or organizational challenges were explicitly addressed. Our interviews were complemented and informed by an analysis of collaboration-internal policy documents, as well as informal exchanges with active and former ATLAS physicists.

## Three strategies

In this section, we introduce and discuss three core strategies of how research collectives deal with organizational complexity. We have identified these strategies through an iterative process, moving back and forth between in-depth analysis of our empirical data and existing literature. This includes organizational theory as well as STS studies into Big Science and its complex organization in fields such as molecular biology and particle physics. We found that selected insights from existing research are compatible with our empirical findings, which, however, also reveal hitherto unnoticed aspects.

The three strategies of researchers attending to organizational complexity are the following: segmenting research infrastructure (4.1), introducing elements of bureaucratic governance (4.2), and implementing standards and standardization (4.3). As we argued above, we understand organizational complexity to be associated with the existence of multiple and overlapping logics of organizing. We propose that each of the three strategies relates to, and interferes with, these logics in distinct ways. Drawing on material from our case study, we will show that the segmenting of research infrastructure separates organizational logics; bureaucratic governance sorts modes of organizing and introduces priorities in their handling; standards and standardization assist in mediating between modes of ordering.

While these strategies, at first sight, seem to reduce organizational complexity, it is important to note that complexity is also being displaced and reappears in various guises elsewhere. A straightforward transformation from a state of complexity to one of simplicity or a single order does not exist (cf. also Mol and Law 2002). What has been ordered in one moment may reappear as unordered, generating novel kinds of complexity, in the next. There thus exists a “possibility of recomplexification” (ibid., p. 13) with the result that ‘simplifying’ processes may have ‘complexifying’ effects.^11^

### Segmenting research infrastructure

Asking how Big Science research attends to organizational complexity, we first focus on a social configuration that is common today: the organizational segmenting of central technical infrastructure from the conduct of experiment, accompanied by a clear division of labor and responsibilities between infrastructure centers and experimental teams. For example, in particle physics, the collider is constructed, run, and maintained by CERN, acting as host laboratory, while experiments are conducted by collaborations (e.g., ATLAS, CMS). Each collaboration is responsible for building, maintaining, and operating its detector, enabling its members to measure and analyze the properties of colliding particles and their decay products.^12^

Such separation of central infrastructure from experiments is typical for Big Science research but also exists in some other fields (e.g., nanoscale research). In the STS literature, centers for research infrastructure of this type are also discussed as user facilities or technological platforms.^13^ Besides particle physics colliders, examples are synchrotron radiation facilities, neutron generators, free-electron laser sources, large telescopes, and cleanroom facilities. A common feature of these facilities is that they provide their services to various experiments run by national or international teams. In the case of cost-intensive, large-scale research infrastructure, this set-up affords resource-efficient sharing of instrumentation and associated expertise. However, we are more interested in another feature of this segmentation: how it relates to organizing practices and, in particular, the organizational complexity of the collaboration.

For the case of physics, it seems rather atypical that a team of experimental scientists would not construct its own research apparatus. In contrast, for example, to life scientists who often work with devices off-the-shelf, physicists take great pride in building their instruments, i.e., the instruments generating the prime data for their analysis. It thus seems noteworthy that experimental particle physicists would have agreed to ‘outsourcing’ one of their two most important and intriguing instruments: the collider (the other instrument being the detector). Designing and building colliders such as the LHC requires not only cutting-edge engineering knowledge but also relies on dedicated research in various areas of physics (e.g., electromagnets, materials, superconductivity).^14^ In this sense, the collider is an epistemic object in its own right, not a ‘mere’ instrument.

For LHC collaborations, however, the segmenting of all collider-related work can be interpreted to be beneficial as it allows the collaboration to focus its attention predominantly on the detector (and associated work). Bracketing the collider’s intricacies and related concerns in all respects – technical, financial, regulatory, political, epistemic, etc. – helps the collaboration contain organizational complexity within the confines of detector-related work. Knowledge and expertise required to produce path-breaking results in an experiment like ATLAS is widely diversified in and of itself, even without considering specialized knowledge of collider technologies. Physicists resolve problems involving theoretical particle physics, detector technologies, trigger and data acquisition, software and computing, physics analysis, etc. This wide range of expertise generates organizational complexity of its own, the collaboration dedicating continuous attention and great care to afford mutual understanding across its heterogeneous expert communities.

Our interpretation is consistent with Knorr Cetina’s (1999, p. 56) observation that collaborations and their members are preoccupied “with the experiment itself, *with observing, controlling, improving, and understanding its components and processes*” (highlights as in original), a feature the author calls the collaboration’s “care of the self” (1999, Chap. 3.4). While the detector and its behavior play a crucial role in the process of self-understanding, the collider does not.

Thus, in terms of the organizational structure, collider work has been pulled apart from the collaboration’s detector-related work. At the same time, in practice, much more is going on. Close cooperation and continuous consultation between collaboration members and collider experts are essential to afford smooth operation of data taking and a proper understanding of the collider’s performance. Such cooperation and consultation processes that physicists have reported to us include the following examples. Collider experts require the assistance of the collaboration when testing different beam configurations in machine development periods. Once the collider is up and running, collaboration and collider representatives jointly participate in the day-to-day coordination of operation runs. Representatives of the collaboration and the collider also consult and mutually coordinate when to interrupt the collider for maintenance and minor repair work.

Within the collaboration, a dedicated unit is responsible for contact and cooperation in the various forms just described. The Executive Board, which leads the implementation of the ATLAS project, includes a ‘technical coordinator’ who is responsible, among others, for the ‘machine interface’ (i.e., the interface with the collider) and the ATLAS infrastructure at CERN. A senior staff of the collaboration explains how the technical coordinator acts as a point of contact:

CERN is the host lab, so is providing the infrastructure [to the collaborations] but has no influence on who will be the spokesperson of this collaboration and how is the internal structure. There are only a few rules […] and the rule is that both the technical coordinator and the resource coordinator [of the collaboration] has to be CERN staff. […] It all relies on CERN infrastructure help and exchanging information, knowledge, resources between CERN and the collaborations. (Interview 2, senior staff, ATLAS)

The work concerned with adjusting the collider system’s and the experiment’s needs is a group-spanning activity that involves not only the technical coordinator but extends across and beyond the collaboration. It has its own complex configuration. Without going into detail, we also wish to mention that alternative cooperative arrangements arise in the preparatory phases of collider and experiments. In this early period, the characteristics of a potential future collider are explored by collider experts together with detector and physics analysis experts (e.g., in the context of the deliberation process to design a ‘European Strategy for Particle Physics’).

To sum up, the segmenting of research infrastructure attends to organizational complexity by keeping distinct epistemic aims and related organizing logics clearly separated. In this case, it builds on an institutionalized form and its associated division of labor, with separate administrative units responsible for the collider and the detectors. However, at the level of everyday practices, we do not only find manifestations of this particular response, but actors’ attempting to bridge, again, the boundaries thus introduced, which may cause organizational complexity of its own.^15^ In other words, actors engage both in organizational segmentation or specialization *and* in smoothing out any associated mismatches between different administrative units.

### Introducing elements of bureaucratic governance

STS scholars have shown considerable interest in the working and configuration of particle physics collaborations. In particular, they have emphasized the communitarian and non-hierarchical nature of these social forms, and associated the collaborations’ governance with consensual decision-making and problem-oriented flexibility (e.g., Boisot 2011; Knorr Cetina 1995, 1999; Shrum et al. 2007).^16^ However, in our interviews, collaboration members typically characterize the ATLAS collaboration as ‘bureaucratic’. In the following, we will consider these contrasting views in more detail. Based on observations from our empirical investigation, we will then discuss the collaboration’s increasing reliance on written rules (formalization) and introduction of novel decision-making bodies. We consider these phenomena to indicate bureaucratization processes and suggest that they can be understood as responses to organizational complexity.

In one of the first uses of the notion ‘Big Science’, the then-director of the Oak Ridge National Laboratory expressed his concern that the growth and industrialization of research would lead to the “subordination of individual scientists under large and bureaucratic projects” (Weinberg 1967, p. 9; cited in Cramer et al. 2020, p. 8) with detrimental consequences for academic freedom and creativity. Such negative connotation of bureaucracy as limiting individual autonomy stems from an ambiguity inherent in Max Weber’s (1978 [1968]) definition. Weber described bureaucracy as the most rational and efficient form of governance, but also as a type of administration where authority resides in hierarchical positions and is enforced through the execution of formal rules and regulations. Especially *formalization* (defining and executing procedural rules) is considered a core element of bureaucratic governance, with both its coercive and enabling effects on individuals and organizations (Adler and Borys 1996).

Despite being a typical example of Big Science, high-energy physics collaborations have long been characterized as being exceptionally void of bureaucratic structures. In a comparative study of research collaborations in several scientific fields, Shrum et al. categorize particle physics collaborations as ‘participatory collaborations’ (Shrum et al. 2007): characterized by flat hierarchies, collective decision-making, an egalitarian status of members, and reliance on verbal agreements or non-binding memoranda, rather than formal contracts. In this reading, particle physics collaborations are thus the opposite of what Shrum et al. view as ‘bureaucratic collaborations’. Similarly, Knorr Cetina describes particle physics collaborations to be void “of hierarchical structures and formal organization, without external supervision and hard-set internal rules” (Knorr Cetina 1995, p. 124). These collaborations would neither exhibit the industrial-style division of labor nor the centralized control expected as a precondition for the success of such a large-scale technical effort. From the perspective of management studies, the ATLAS collaboration is seen as an ‘adhocracy’, a network of experts with distributed decision-making authority (Boisot 2011). Adhocracies organize work in projects and are managed by experts (not administrators) in a task-oriented, ‘ad-hoc’ manner, which makes them both more innovative and less efficient than bureaucratic organizations (cf. Mintzberg 1979). Emphasizing the non-hierarchical organization of ATLAS and the low degree of codification, Boisot (2011) sees the collaboration as lacking centralized control and abstract coordinative structures, resembling a ‘clan’ rather than a ‘bureaucracy’.

Our case study builds on the hypothesis that recent transformations in particle physics – present collaborations being much larger, more diverse, and more international than their predecessors – are observable in the practices of organizing. In our interviews, ATLAS physicists repeatedly emphasized an increase in the formalization of decision-making procedures and the number of organizational levels and positions introduced to manage these procedures. In earlier experiments, decisions would have been based on simple votes and informal face-to-face deliberations, whereas now, extensive protocols would be followed:

While [in previous experiments] we said, ‘we need a new physics coordinator, we thought it could be this or that person’, we exchanged opinions, and in the end there was a vote, but here [in ATLAS] this is a proper election, there is a search committee that is doing a search, then they are filtering out candidates, then afterwards three candidates are proposed for election, and then there is a proper election. Just like in parliament. (Interview 25, senior scientist, ATLAS)

In addition, the number of coordinators and committees overseeing a given workflow, such as the preparation of new results and publications, is said to have increased. While physicists tend to characterize the many approval steps as cumbersome, they also acknowledge the important ordering function of formal procedures and documentation:

In ATLAS, you have all this structure and bureaucracy that keeps everything in place. (Interview 28, former ATLAS Ph.D. student)

The effects of the organizing practices that our interviewees labeled as ‘bureaucratic’ thus are not straightforward; they may be described as time-consuming and restrictive, but at the same time also as enabling more efficient collaborative work through collective oversight, tighter integration of distributed work, and reduced internal competition.

As indicated above, the introduction of formal procedures in the collaboration goes hand in hand with setting up novel committees to oversee and implement these. An example is the complex task of selecting speakers for talks at international conferences, which is handled by two separate committees. A first committee is responsible for the selection proper, and a second – the *Speaker Committee Advisory Board* – assists this process by building and maintaining a database of potential speakers. The advisory board also reviews the overall procedure and ensures that the distribution of talks among collaboration members is fair and adheres to the collaboration’s guidelines. Conference speakers are selected according to several criteria of eligibility, which feed into a formalized ranking.

In her study of the earlier CERN experiments, Knorr Cetina (1995, p. 132) portrays the procedure of speaker selection as taking place during a collaboration meeting in an ad-hoc and informal manner. The formalization of processes and delegation of decision-making authority to specifically appointed bodies within the collaboration that we described above is thus a recent phenomenon and, as we suggest, reflects the increased organizational complexity of LHC collaborations. One may wonder whether this new mode of governance threatens the collaboration’s professed egalitarian constitution. We do not think so. Instead, we would view it as a strategy of the collaboration to arbitrate between different organizational logics: the logic of egalitarian, participatory self-governance and the logic of efficient and transparent administration – of “keeping everything in place” (interview). Elements of bureaucratic governance, such as formal procedures and decision-making authorities, may actually assist the collaboration in *maintaining* its participatory and consensual governance in the face of a growing and diversified membership. Where informal face-to-face communication and ad-hoc collective decision-making involving all collaboration members are impossible to realize or would result in unfair advantages for collaboration members with stronger informal networks, such processes are delegated to specifically appointed committees and facilitated by formalization. The decision-making process is still deliberative since committees are expected to reach consensual decisions. It also remains egalitarian and participatory in the sense that committee members are appointed for a fixed period only, and these rotating positions are open to all collaboration members.

Through formalization and the accompanying introduction of decision-making bodies with clearly circumscribed jurisdiction, the collaboration facilitates internal governance and simplifies particular tasks, in this sense, reducing organizational complexity. Yet, this process may also generate new complexity when unforeseen situations escape the rules of due process and engender further attention and effort. An example of this effect is the speaker selection process. As physicists told us, it regularly occurs that none of the eligible candidates is willing to actually attend the proposed conference, or that the pool of qualified speakers for a niche topic is exhausted. In such cases, the formalization of the speaker selection process generates a need for further informal communication and coordination efforts within the collaboration; official rules need to be side-stepped and additional principles, such as an appeal to higher authority (asking the management to approve an alternative candidate), may have to be invoked.

To sum up, in our view the discussed agreements and procedures are the collaboration’s self-reflexive response to coordination issues arising from the growth of the collaboration, which require some amount of managerial oversight, *and* the perceived need to ensure participatory self-governance. However, the elements of bureaucratic governance introduced to solve this tension between opposing organizing logics are themselves frequently challenged and sometimes circumvented.

### Implementing standards and standardization

Apart from the segmenting of research infrastructure and bureaucratic formalization, we have identified a third strategy of how researchers and their collectives address organizational complexity: standardization. The notion refers to the creation of a standard (a standard unit, procedure, size, etc.) as well as to the alignment of practices and objects following a standard’s implementation. One creates standards typically “with the aim of obtaining legitimate coordination, comparability, and compatibility across contexts” (Timmermans and Epstein 2010, p. 75). Accordingly, standards and standardization are frequently described as means to facilitate collaboration across disparate entities, among others in research collaborations. From an epistemological perspective, standardized methodologies support collective accountability insofar as a central coordinator could in principle understand and integrate all the individual contributions to a research process (Huebner et al. 2017). As close analyses of standards and standardization practices in STS show, standards do not work by themselves, but they are built upon supportive infrastructures, enforced or incentivized by social measures, and, at times, adapted to changing circumstances (Bowker and Star 2000; Timmermans and Epstein 2010). Standardization practices participate in “screen(ing) out diversity” (Star and Lampland 2009, p. 8) by enacting or highlighting one set of (aesthetic, epistemic, moral, etc.) values rather than another. In this sense, the standardization of practices and objects can be seen as reducing complexity, but it comes at the expense of suppressing alternative practices and objects. Thus, the same standard may be experienced to be facilitating or constraining.

In view of their potential to facilitate coordination across contexts, standardization processes have been analyzed as to their effect on the configuration of new research fields and communities (cf. Merz and Sormani 2006). For the case of cancer research, Fujimura (1996; 1992) shows that a set of standardized research materials and technologies from molecular biology was introduced into the diverse and interdisciplinary research landscape together with the theory of proto-oncogenes (i.e., the theory that mutations in specific human genes cause cancer). This ‘standardized package’ of concepts and research tools afforded the consolidation of cancer research around a single theory. The ‘screening out’ of conceptual and methodological diversity helped reduce the complexity of the phenomenon under study and align the research field with the new standard approach from molecular biology.

Another case for the importance of a standard is made by Hilgartner (2017, Chap. 4). He argues that a particular standard for reporting data, the ‘sequence-tagging site landmark’, assumed an essential role for the Human Genome Project, the large-scale effort to sequence the human genome. Where data were reported in this standard, maps of the genome produced by different laboratories could be integrated and combined, and new results were easily shared within the worldwide scientific community. The sequence-tagging site standard was also used to evaluate the performance of genome centers in terms of the number of maps produced (ibid.). Standardization thus facilitated collaborative work across independent research units and simultaneously offered a measure for setting shared goals and monitoring productivity. Both effects were essential in re-organizing a complex institutional research environment, i.e., a distributed scientific community, where a common standard mediates between diverse units and organizational logics.

Such re-organizing of a research environment through standardization also occurs at CERN. We observed the ATLAS collaboration to have created and implemented a range of standards concerning the epistemic and organizational dimensions of diverse work areas. There exist standard software and frameworks, standard tools for data analysis, and standard procedures for workflows such as editing a publication. In the following, we focus on two examples where standardization facilitates coordination across the ATLAS collaboration: the standardization of research tools and the introduction of a standard unit for the measure of ‘service work’.

The *first* example concerns the implementation and maintenance of standardized data analysis tools, such as the algorithms for the reconstruction of specific objects in the detector. In the collaboration, ‘combined performance groups’ ensure that the algorithms’ performance and limitations are well understood. The groups test the algorithms for their efficiency in identifying the proper objects by comparing the results between simulated and real data. Sharing standardized analysis tools across the collaboration has the advantage that the physicists conducting the actual analyses do not need to develop their own tools for each search, and that the epistemic uncertainties involved in using a specific algorithm are minimized. The standardization of tools reduces task complexity by limiting the range of the analysts’ possible approaches and “the amount of tacit knowledge, discretionary decision-making, and trial-and-error procedures needed to solve problems” (Fujimura 1992, p. 179, footnote). As a collaboration member put it:

[With a standardized tool] it’s easier to solve issues and you avoid everyone going through the same problems from start to finish. (Interview 22, senior scientist, ATLAS)

In this sense, standards make research tools accessible to a wider range of users, they streamline epistemic processes and reduce their complexity. Insofar as standardization indicates the reliability of tools and the comparability of results coming from different groups and analyses, it also has a crucial function in securing the credibility of research within the collaboration and beyond.

However, the standardization of analysis tools also has disadvantages, particularly in situations where the available tools do not yield the best possible result. In this case, it can be challenging to follow an alternative approach as a Ph.D. student experienced who had developed a new algorithm for a specific analytical task that outperformed the standard procedure:

When I looked at the results [based on my new algorithm], they were almost twice as good as those coming from the standard tool. […] But then I heard from other groups that there is a reason that this isn’t standard yet, because it is more complicated than the current standard. (Interview 10, Ph.D. student, ATLAS)

A novel approach, although “almost twice as good” in terms of results, may still be rejected by the working group.^17^ Group coordinators weigh the chance of having an improved result against the delay incurred by extended testing of the new approach, when the use of standardized tools promises faster publication.^18^

Although individual collaboration members criticize these constraints on novel approaches, they also emphasize that the standardization of epistemic contributions and processes assumes an important ordering function within the collaboration:

But again, this is all needed in order to ensure that all the scientific methods are correct and that all publications meet a certain standard of quality. (Interview 5, post-doc, ATLAS)

Not getting one’s novel analysis tool through due to extensive approval processes appears to be acceptable in view of a common standard of quality that all contributions and approaches need to meet. Standardization of research tools may thus be understood as a response to organizational complexity insofar as it reconciles an organizational logic of enabling independent work in small teams with practices adhering to the logic of collective ownership and review of results.

Our *second* example, introducing a standard unit for measuring contributions to the maintenance of communal infrastructure, illustrates how collectives use standards to monitor and enable an adequate distribution of shared responsibilities. The ATLAS collaboration refers to contributions to the operation and maintenance of the experiment’s hardware and software as ‘operation tasks’ or ‘service work’, more colloquially. In earlier experiments, which were smaller, this work had been distributed informally based on volunteering. Today, service work is measured in so-called OTP (operation task planning) credits. Each member group^19^ is expected to fulfill a specific quota of service work in terms of OTP credits per year, calculated proportionally to the institution’s number of ATLAS authors, “such that all the onerous tasks are evenly distributed” (interview 13, Ph.D. student, ATLAS).

A researcher can obtain OTP credits in various ways, e.g., through taking shifts in monitoring the detector operation, contributing to technical upgrade work, engaging in software maintenance, or taking on a coordinator role within a group or committee.^20^ By making such heterogeneous contributions comparable and affording their aggregation, the OTP standard allows researchers and groups to hold each other accountable to the same measure of productivity. As a result, the organizational task of fairly distributing ‘service work’ is simplified. However, and importantly, this does not mean that service work is subject to completely centralized control. Once a standard exists, the responsibility to meet the quota resides with the member groups. Standardization in this case facilitates the self-coordination of collaboration members – the member groups can still find individual internal solutions for the required service work – and presents an alternative to centralized solutions.

In addition, we see that standards also serve as the building blocks of formalization practices in ATLAS described above. An individual’s OTP score, for example, informs a researcher’s ranking and the likelihood of his or her selection as a potential conference speaker:

When you do service tasks, like different shifts and stuff, you get these operational points, they’re called ‘OTP’, and your ability to give a talk is based on, how many points you have basically, and how long it’s been since the last time you gave [a talk]. (Interview 16, Ph.D. student, ATLAS)

The use of standardized criteria to produce a ranking of eligibility reduces the complexity of the speaker selection process by making a very heterogeneous pool of candidates readily comparable. A metric such as the OTP, however, necessarily emphasizes some values over others (Espeland and Sauder 2007). As long as OTP credits are considered an adequate measure of contributions, the OTP credit system ensures that service work is equally distributed. Tensions surface where tasks do not easily fit within the range of the OTP system, as seems to be the case for software maintenance:

One of the problems with OTP was that for software, at one point they were like, ‘really people are earning more OTP than we can give out’ […] and so they’d say like, ‘Well just know that there’s only so much OTP that you’re allowed to ask for even if you really qualified for more’. (Interview 18, Ph.D. student, ATLAS)

Following this account, the standard credit system misrepresents the actual amount of service work performed on the software infrastructure of ATLAS, which contributes to the interviewee’s personal impression that this kind of work is insufficiently recognized in the collaboration. To meet the increasing demand for software maintenance work in ATLAS, a more fine-grained credit and reward system might have to be developed, bringing back in the diversity that was initially screened out in the OTP system, and adding another layer of organizational complexity.

To conclude, common standards and standardized practices relate to organizational complexity insofar as they mediate between different organizational logics. Rather than fixing in place certain procedures, they allow different practices to co-exist as long as they can be framed within the same standard. However, since standards “screen out diversity” (Star and Lampland 2009, p. 8), they never fit all the logics and practices within an organization. Where standards are experienced as inadequate for ensuring comparability and compatibility, they are at risk of losing their collectively binding character and instead contribute to the proliferation of work in need of ordering, thus generating novel organizational complexity.

## Conclusions

We introduced and discussed organizational complexity in the context of Big Science research focusing on actors’ practices and strategies in attending to their sociotechnical environments. We identified these strategies based on literature on the organization of large-scale research and findings from our empirical study of the ATLAS collaboration. Our concept of organizational complexity, which refers to scientists’ organizing practices, adds an additional perspective to existing literature that has focused on complexity as an effect of organizations’ size and structure or interpretation and representation. We suggest that organizational complexity manifests itself where organizing practices follow and enact different logics. Research collectives such as the ATLAS collaboration may be more or less complex depending on the degree of organizational reflexivity, i.e., the extent to which the practices and logics of organizing receive actors’ attention. We suggest that this reflexivity may increase over time in large-scale research, where actors must not only secure scientific success and technological innovation, but also sustain appropriate organizational forms in the long term.

We described how actors in large-scale research address divergent organizational logics in practice. We discussed three partly overlapping strategies that research collectives employ to cope with organizational complexity: segmenting research infrastructure, introducing elements of bureaucratic governance, and implementing standards and standardization. The results of our case study illustrate that these strategies, when implemented in practice, do not have straightforward effects.

Ceding the organizational and epistemic responsibility for the particle collider to the CERN laboratory allows an experimental collaboration to focus on the detector. This *segmenting of infrastructure* and responsibilities between the laboratory and the ATLAS collaboration divides the aims (long-term maintenance of collider infrastructure vs. experimental program) and logic of the two communities. This divide, however, needs to be organizationally bridged by installing points of contact between them to achieve coordination and integration despite segmentation.

Within the collaboration, introducing elements of *bureaucratic governance* delegates the work of deliberation and decision-making to designated members. Implementing formalized procedures and decision-making responsibilities arbitrates between the competing logics of participatory self-governance and efficient (also transparent) management of internal processes. However, while this purpose appears to be achieved, as bureaucratic measures allow for “keeping everything in place” (interview), this strategy itself remains necessarily incomplete and a matter of internal contestation. As a result, regulations must be regularly updated, and the work of formalization is never complete.

Finally, *standards* introduced for research tools and monitoring shared maintenance responsibilities facilitate coordination in meeting collective goals. The standards address complexity through reconciling two distinct logics: individual merit (the pursuit of novel ideas and the recognition of contributions to shared infrastructure) is considered important by members of the collaboration, but commitment to collective ownership, responsibility, and oversight is regarded as crucial, too. In making diverse practices and contributions comparable, standards, however, emphasize some qualities over others and may generate unintended effects where they become inadequate. To retain their collectively binding character, standard tools and organizational standards need to be frequently adapted or even circumvented.

Altogether, our main contribution points to a basic ambiguity. Every strategy of coping with organizational complexity, once put into practice, generates new challenges, including the unintended creation of further complexity. Attempts of taming complexity thus displace complexity and make it reappear elsewhere. In other words, we highlight the process-like, dynamic nature of organizational complexity. It is impossible to reduce or eliminate complexity once and for all. Instead, attending to complexity is an ongoing concern. One can witness this in the ATLAS collaboration, which continues to invent and modify procedures and establishes dedicated committees and coordinator roles to tackle organizational complexity, even years after it has begun data taking.

We have discussed only some of the logics associated with increasing organizational complexity in the ATLAS collaboration, leaving out others. To name a few, we did not address the different institutional affiliations of researchers (in countries across the world) with their corresponding norms, the diversity in the researchers’ national and cultural backgrounds, and the associated strive for adequate representation, or the differing priorities and prestige of research topics within the collaboration. We do not assume all of these organizational challenges to be attended to by the three strategies described but would expect that additional organizational responses can be identified in the ATLAS collaboration and in other examples of large-scale research. Similarly, we believe the specific strategies and practices associated with organizational complexity to vary across research fields, infrastructures, or sociotechnical configurations. We hope that further studies may use our approach to investigate how practices and strategies associated with organizational complexity shape scientific knowledge and knowledge production in specific ways.

## Data Availability

Not applicable.

## Footnotes

1 This contribution was developed from a presentation delivered at the interdisciplinary conference *Simplicities and Complexities* that took place in Bonn in May 2019. Following the invitation of the editors, we provide a take on complexity and simplicity that is explicitly rooted in scholarship from the social studies of science and might thus be considered complementary to debates in philosophy of science.

2 Clarifying the notion of complexity is a notoriously difficult task. Despite the frequent appeal to complexity as a defining feature of living organisms, for example, biologists as well as philosophers of science tend to eschew definitions of what exactly makes an organism or their representation more or less complex. For example, Gross (2021, this volume) argues that the notion of complexity captures diverging intuitions, as it may both refer to the emergence of complex patterns of behavior from simple interactions, as well as the intricacy of a system consisting of heterogeneous elements interacting in a non-simple manner.

3 Cf. e.g., Hallonsten (2016), Cramer (2020), Cramer and Hallonsten (2020). For a conceptual analysis of Big Science, cf. Vermeulen (2009).

4 This research has been conducted in the context of the interdisciplinary Research Unit *The Epistemology of the Large Hadron Collider* and its sub-project ‘Producing Novelty and Securing Credibility: LHC Experiments from the Perspective of Social Studies of Science’.

5 Due to our focus on organizational complexity, the complexity of technical infrastructure moves into the background. We also cannot provide a detailed discussion of the myriad challenges that scientists face in conceptualizing, reconstructing, and representing their objects of inquiry. However, with our foregrounding of organizational practices we do not mean to imply that technical, epistemic, and organizational dimensions of research can be clearly separated. Rather, we assume that divergent logics and modes of ordering run through all dimensions of research. Organizational complexity, as we see it, is an expression of technical and epistemic complexity.

6 In organizational research, such understanding is known as a ‘relational or process conception’ of organization (Scott 2004) and is closely related to the work of Weick (1969).

7 A rich resource to rethink notions of complexity from a practice perspective is the volume *Complexities: Social Studies of Knowledge Practices*, edited by Law and Mol (2002) and in particular its introductory chapter (Mol and Law 2002).

8 Law emphasizes that modes of ordering should not be understood as temporally or ontologically prior to the practices that embody them. Similarly, we do not assign ontological priority to either logics or practices.

9 For particle physics experiments, Knorr Cetina similarly speaks of a “constant humming of the experiment with itself, about itself” (Knorr Cetina 1999, p. 173).

10
https://atlas.cern/discover/collaboration (seen October 1, 2021).

11 Knorr Cetina (1999) portrays a related notion of complexity, asking how complexity emerges in practice. She sees it as resulting “from the interference and blending of locally configured and anchored orderings” and as associated with “the creation of second- and third-order structures” interdependent with “the original level, creating complex tangles” (ibid., pp. 245–246).

12 Organizationally, a collaboration of physicists can propose a new experiment to CERN. The proposal is then reviewed and approved or rejected by the CERN Council. However, CERN is not fully funding or operating the experiment if approved. Instead, the institutes and countries involved in the collaboration will fund the experiment.

13 For an overview, see Cramer and Hallonsten (2020), D’Ippolito and Rüling (2019), and Hallonsten (2016).

14 This research is published in journals such as *Nuclear Instruments and Methods in Physics Research Section A: Accelerators, Spectrometers, Detectors and Associated Equipment*.

15 This is an interesting contrast to research infrastructures such as synchrotron radiation facilities, where the social and organizational separation between lab and experiments is more pronounced and durable.

16 Perović (2018) criticizes that these accounts promote an idealized image of high-energy physics organizations as exceptionally egalitarian, although egalitarian self-organization of experts is not unusual in knowledge-intense bureaucracies. However, since Perović does not distinguish between the organization of laboratories and the organization of experimental collaborations, it remains unclear whether the ATLAS collaboration could be considered a bureaucracy in his sense.

17 These ideas and novel approaches are not necessarily lost. Ph.D. students, for example, may develop and discuss new techniques in their dissertation, although these are not included in an official ATLAS publication.

18 This is an example of a trade-off between the speed and reliability of results in non-application-oriented research. For a discussion of the ethical and epistemological aspects of time-sensitivity in fundamental and applied research, see (Jadreškić 2020).

19 ATLAS is the joint effort of member groups formally affiliated with university departments or research institutions, each of which contributes part of the budget to the experiment’s operation.

20 These credits are also documented on an individual level.
